# Illinois State Veterinary Medical Association

**Published:** 1894-12

**Authors:** Matthew Wilson

**Affiliations:** Secretary; Mendota, Ill.


### ILLINOIS STATE VETERINARY MEDICAL ASSOCIATION.

The twelfth annual meeting of the Illinois State Veterinary Medical Association was held at the Sherman House, Chicago, November 26th and 27th, 1894. The meeting was called to order at 2 P. M., November 26th, by the President.

The roll being called, the following members responded to their names:

Drs. A. G Alverson, A. H. Baker, F. L. Brown, A. Babb, L. Campbell, V. E. Frizzel, J. T. Nattress, C. E. Sayre, Jno. Scott, R. W. Story, F. S. Schoenleber, A. M. Story, M. R. Trumbower, J. H. Ullrich, J. L. Tyler, M. Wilson, R. G. Walker.

The minutes of the last regular meeting were read and approved.

The President's annual address was then listened to.

The Treasurer being absent from last regular meeting, was unable to make a complete report.

The reports of the standing committees were received, and the committees discharged.

Drs. Gunning and Newby being absent, but having sent their papers, they were read by the Secretary.

Dr. R. W. Story was then called upon for his paper on "Acute Indigestion."

The usual lengthy discussion of the disease followed.

The meeting adjourned until November 27th, at 10 A. M.

November 27th.—Upon reconvening, the President called upon Dr. M. R. Trumbower, the State Veterinarian, who spoke on Actinomycosis, and the methods pursued in the inspection of meats at the Chicago Stock Yards.

This was followed by many remarks from the members on the methods that ought to be pursued in the inspection of meats in the country, for local consumption, also on the dangers arising from the use of flesh and milk from diseased animals for human consumption.

Dr. Tyler was then called upon for his paper on "Omphalo Phloebitis."

The meeting then adjourned until 2 P. M.

Upon reconvening, Dr. C. E. Sayre was called upon for his paper on "Homeopathy in Veterinary Practice."

This paper brought forth quite an animated discussion, but was ably defended by Dr. Sayre from his standpoint.

Dr. S. S. Baker then read his paper on "Remarkable Cases in Practice," and was followed by Dr. Campbell in his paper on "The Unexpected Recovery of a Case of Sunstroke"

The following gentlemen's names were then proposed for membership: Dr. Harry G. Hoover, "Am. V. C., '94," Sterling, Ill., vouched for by Dr. Trumbower; Dr. Chas. W. Johnson, "Ch. V. C., '87," Elburn, Ill., vouched for by Dr. A. H. Baker; Dr. E. L. Quitman, "Ch. V. C., '91," Chicago, Ill., vouched for by Dr. A. H. Baker; Dr. W. G. Neilson, "Tor. V. C., '91," Monmouth, Ill., vouched for by Dr. R. W. Story.

A motion was made by Dr. Wilson, seconded by Dr. Baker, that the above named gentlemen be elected to membership by acclamation. Carried.

The election of officers resulted in the following named gentlemen being elected for the ensuing year:

President, Dr. M. Wilson, Mendota, Ill.; Vice-President, Dr. A. G. Alverson, Bloomington, Ill.; Secretary, Dr. A. Babb, Springfield, Ill.; Treasurer, Dr. R.W. Story, Princeton, Ill.

Board of Censors: Drs. C. E. Sayre, M. R. Trumbower, J. T. Nattress

Dr. Wilson taking the chair, Dr. Trumbower made the suggestion that a new association be formed consisting of members in the Mississippi Valley.

Remarks in favor of such an association being formed were made by various members, and a motion made by Dr. S. S. Baker, and seconded by Dr. Trumbower, to the effect that the Secretary be instructed to correspond with the secretaries of associations in surrounding States regarding the formation of such an association, was carried.

The question of legislation regarding the introduction of a bill, in the Legislature, to regulate the practice of Veterinary Medicine and Surgery in this State was next discussed.

It was found that the members of this association present were in favor of the introduction of such a bill, and a motion made by Dr. A. H. Baker, seconded by Dr. Scott, that the sum of $10.00 each be subscribed to defray expenses of introducing such a bill, was carried.

Dr. Baker moved, and seconded by Dr. Scott, that the Chair appoint a committee of three to be known as the Committee on Legislation, and such committee be given power to act. Carried.

The chair appointed as such committee, Drs. S. S. Baker, Jno. Scott and J. T. Nattress.

Bills to the amount of $30.85 were audited and ordered paid.

The following standing committees were then appointed: Committee on Program, Drs. S. S. Baker, J. T. Nattress, T. J. Gunning; Committee on Arrangements, Drs. Jno. Scott, A. H. Baker, M. R. Trumbower; Committee on Membership, Drs. J. L. Tyler, A. M. Story, W. J. Martin.

A vote of thanks was tendered the retiring officers for efficient services, also to the essayist, and to the proprietors of the hotel for their accommodations.

The meeting then adjourned to come together at the call of the committee, in Peoria, next February. MATTHEW WILSON, M.R.C.V.S., *Secretary.*

MENDOTA, ILL.

